# Documenting the evolution of the relationship between the pharmacy support workforce and pharmacists to support patient care

**DOI:** 10.1016/j.sapharm.2016.10.012

**Published:** 2017

**Authors:** Tamara Koehler, Andrew Brown

**Affiliations:** a University of Applied Sciences, Netherlands; b A.N.Brown Health Systems Strengthening Consultancy Pty Ltd, 121/54 Printers Way, Kingston, ACT, Australia

## Abstract

Since 2009 there has been a focus on the relationship between pharmacy technicians, pharmacy support workforce cadres and pharmacists in the literature. 2009–2011 saw a framework of role evolution develop, with publications from 2012 to 2015 documenting further maturity in the development of practice models for improved patient care and optimal use of personnel. The dominant narrative in the published academic literature has been made by certain high- income countries (mainly Canada, Denmark, United Kingdom and the United States of America). In these countries there are significant numbers of pharmacists available and there has been an increasing interest to utilize pharmacy support workforce cadres to allow the extension of clinical roles of pharmacists in these contexts. This is not a systematic presentation of all the literature available but rather a commentary overview supported by key papers. Key points from this literature include:

• *The initial interest in this area resulted from a growing desire to increase the visibility of the pharmacist in line with the evolution of pharmacy practice, investigating how to move the pharmacist away from administrative roles and more towards clinical care. In this context the optimal use of other pharmacy cadres has gained momentum with numerous examples provided where pharmacists have been able to extend their clinical role with greater use of pharmacy technicians or other pharmacy support workforce cadres.*
• *As an expanded role for a variety of pharmacy support workforce cadres has developed, issues around education, regulation and registration have been discussed. As has the importance of clearly defining the role of both the pharmacist and other pharmacy cadres in specific practice settings. Where these partnerships have been developed successfully there has been detailed attention to change management and stakeholder engagement, with improved patient care as the focus. Significant guidance has been provided to aid implementation.*
• *In more recent years, pharmacy support workforce roles have expanded in some country practice settings, moving from administration and supply functions to independent checking of prescriptions by cadres and more recently to the management of patient adherence programs (e.g. United Kingdom, USA).*
• *With a continued focus on freeing up the pharmacist for expanding clinical roles, the most recent literature (USA), explores the need to further develop the leadership skills of the pharmacy support workforce.*

(To allow the reader to clearly understand the country of origin of the themes presented, care has been taken to note the country of origin of the papers used in this commentary).

## 1 Increasing the visibility of the pharmacist starts the discussion on pharmacy support workforce roles and skills mix

The discussion regarding the role of pharmacy technician and pharmacy support workforce roles is strongly influenced by the discussion on the pharmacist's role and the evolution of that discussion from a consideration of core pharmacy roles based within a dispensary, to a discussion of decentralized roles where pharmacists conduct more activities in patient-care areas. According to Horon et al. (Canada), this debate can be seen as a positive indicator of the progress that the profession has made in clarifying the pharmacists role as clinical, to deliver patient-centred pharmaceutical services.^1^

In 2012, this discussion speaks of opportunities to improve the quality of healthcare and continuation of the process in which the profession moves forward from product care to patient care (USA).^2^ It is suggested that in this new environment a different role for pharmacy support workforce cadres is required to complete more administrative tasks to fill the gap created by the transition of pharmacists to more clinical care roles. It is noted that any new pharmacy support workforce roles need to be aligned with the core strategies of the community and hospital pharmacy, to provide a successful pharmacy practice model, based on a sound medicine distribution system that safely and efficiently gets medication to patients (USA).^3^

## 2 The need to explore the changing role of pharmacists & pharmacy support workforce cadres

The literature consistently presents the need to expand the pharmacist's clinical role. Different initiatives have been established to clarify the process of moving the profession forward into the era of clinical care.

From the Canadian perspective, (2008, Canadian Society Hospital Pharmacists (CSHP); a vision of Pharmacy Practice Excellence by the year 2015), *“utilizing well-trained pharmacy technicians is a must, if the profession is to achieve optimal use of the pharmacy workforce and meet increasing expectations for clinical pharmacy”.* This will lead to pharmacists able to concentrate on intellectual decision making and to provide ample patient care and clinical services even though pharmacists are in short supply both nationally and internationally (Canada).^1^

Also in 2008, the literature presented three drivers for this change. The content-based shift (from product to patient care) creates a void, but there is also a functional gap to fill since pharmacists are having to prioritize their services to the sickest patients and those with complex or high-risk medication regimens. As a result, not all patients that could benefit from clinical pharmacy services, receive them (Canada).^1^ At a more basic level, there is often a significant shortage of pharmacists despite their established roles as members of healthcare teams within hospitals and in communities (Canada).^4^ Besides staff shortages to meet the care provision, sometimes pharmacy-based involvement is not present. It is reported to be the case in Australia, where extending all roles of rural healthcare providers is often necessary to improve access to medication services (Australia).^5^ There are many similar experiences in low- and middle- income countries today, where pharmacists are few.^6^ For example; Papua New Guinea, Vanuatu and Malawi.^7^, ^8^, ^9^, ^10^

In 2011 the literature presents medication supply as the most important domain that needs attention to provide pharmacists with time to expand their own roles to patient centered pharmaceutical care (USA).^11^ Additionally, the effects of medicines shortages on pharmacy departments and the healthcare system as a whole can place significant strain on pharmacists (USA).^12^ Optimization of medication delivery is thought to be achieved by automation and highly trained pharmacy technicians.

Besides the CSHP 2015 Initiative other guiding documents present a call for action. The blueprint for pharmacy (2005; Canadian Pharmacists Association; optimal drug therapy outcomes for Canadians through patient-centered care) and the pharmacy practice model summit (2010, American Society of Health Systems Pharmacists), are seen as a basis to encourage hospital and health-system practice leaders to examine how they deploy their resources to ensure that the efforts of pharmacy departments are aligned with the most urgent needs of patients and institutions.^2^ This advancing of the profession calls for new and more advanced roles for pharmacists, pharmacy technicians and other pharmacy support workforce cadres (USA).^13^ They should work to their full scope of practice (Canada).^14^ In 2013, Medication Therapy Management is regarded as one of the emerging fields for these advancements (USA).^15^

From 2014, literature from Canada focuses on how the provision of healthcare becomes increasingly collaborative, as the healthcare needs of patients grow more complicated (Canada).^16^ To improve pharmaceutical patient care in these settings, this collaborative spirit should also occur between pharmacists, pharmacy technicians and other pharmacy support workforce cadres, not just be extended to professionals outside the pharmacy (Canada).^16^ Or even more specifically addressed, other healthcare professions such as nursing and medicine have already, albeit to different extents, embraced the concept of intra-professional collaboration with support personnel to improve patient care, and the profession of pharmacy should not be any different (Canada).^4^ The increasing scope of practice with the expansion of patient care activities, makes the work in a pharmacy more demanding. This warrants workflow modifications in terms of traditional roles and responsibilities in dispensing medications and providing patient care.^16^

Another perspective is presented in a periodical from the United Kingdom, where the expanding roles of pharmacy technicians are noted to provide more time for pharmacists to conduct clinical functions. The article suggests that pharmacists will need to get reimbursed for these roles, otherwise expanded pharmacy technician roles will place more pressure on pharmacists.^17^

## 3 How pharmacy support workforce cadres support the evolving clinical role of pharmacists

Besides explaining the need for the expansion of the pharmacist role, suggestions are also made in the literature on how to fill in the role of the pharmacy technician in order to support the pharmacist. Again, Canada is used as an example where they state that care is to be provided in a collaborative manner where pharmacists focus on medication management and patient health outcomes, and pharmacy technicians focus on medicine distribution (Canada).^14^ In this paper the Canadian authors have a clear view on the specifics of that role. As pharmacists expand their focus from medicine distribution to providing direct patient care, they suggest relying on pharmacy technicians as managers of medicine distribution systems. This is a role pharmacy technicians are capable, willing and ready to do in the Canadian context (Canada).^14^

The demands for supply chain management in rural areas is obviously a strong driver of the role expansion towards drug distribution, as this paper from Australia notes. The benefits of expanding the roles of pharmacy support staff, thereby releasing pharmacists to focus on more advanced roles becomes clear in rural areas. Rural hospitals that do employ pharmacists often do not use a pharmacist's expertise appropriately, limiting their functions to fulfil basic dispensing tasks. So, pharmacy technicians and other pharmacy support workforce cadres can support medication supply processes (under supervision) to provide more effective basic pharmacy services (Australia).^5^

Most literature focusses on ‘medicine distribution’ roles for pharmacy support workforce cadres, but some articles try to see beyond this point. Nonclinical activities are mentioned in the community practice setting, on the requirement of ‘regulated’ pharmacy technicians in Canada. Proposed effects of this revised skills mix include improved professional satisfaction, enhanced patient care opportunities and economic benefits for the pharmacy.^16^

One of the most progressive ideas from the Canadian literature relates to the contribution of competent pharmacy technicians to important elements of clinical pharmacy services, so direct involvement in ‘patient care’. Obtaining medication histories and tracking the results of laboratory tests are seen as elements contributing to a more desirable pharmaceutical services model and subsequently allowing pharmacists to focus on the challenges associated with the ascending multidisciplinary collaborative practice.^1^

## 4 Exploring the readiness of organizations for change and the importance of following good change management practices

Ideas on the expansion of the roles of pharmacy support workforce cadres that deviate from ‘medicine’ invoke the most interest when more expansive roles are discussed. These papers do not address the appropriateness of the proposed expanded role, but mainly the themes of perception by the pharmacist, and organizational readiness for change.^18^, ^19^ This part of the literature adds insights into the change process by defining possible risks and unwanted outcomes.

The first of these presented insights concerns pharmacy support workforce cadres working without supervision (Canada).^1^ Horon et al. stress the importance of using a collaborative model, inside which these cadres should work. They can be deployed to patient care areas to facilitate the pharmacist's delivery of a comprehensive package of clinical pharmacy interventions, while keeping in mind that pharmacy support workforce cadres are meant to support not replace the clinical role of pharmacists. They acknowledge the fact that more pharmacy technicians in patient areas will lead to more public awareness of pharmaceutical services, but warn for the following possible outcomes: stakeholders (patients, healthcare professionals, administration) could mistakenly conclude that activities performed by pharmacy technicians represent the full catalogue, depth and quality of clinical pharmacy services. It is interesting to note that in many low- and middle- income countries pharmaceutical services can only be delivered by pharmacy support workforce cadres due to a lack of available pharmacists, particularly in rural and remote areas.^6^, ^20^

Concerns with regard to organizational readiness were voiced by authors who shared their sense of *‘think before storming off and change’*. A paper from Canada provides a good example.^14^ If you only get one chance at organizational change, proceed with caution to ensure the successful outcome of effective and safe handover of responsibilities. Questions raised in this context were: Does the entire pharmacy profession support the concept of pharmacy support workforce cadres taking on this expanded role? Can healthcare organizations accommodate this change? and on a more day-to-day basis: Are pharmacists willing to support and collaborate with pharmacy technicians? Are pharmacy technicians and other pharmacy support workforce cadres equipped to fulfil those responsibilities? The article provided ways to become well informed and equipped to initiate transition by formally presenting success stories and the sharing of experiences by ‘early adopters’ (Canada).^14^

In further considering readiness for change other examples appear in the literature. The assessment of the effect of entrepreneurial orientation, resource adequacy and pharmacy staffing on pharmacy practice change showed, among other factors, that the most commonly reported change in pharmacies over the previous two years included the responsibilities and activities of pharmacy technicians (USA).^15^ When current and desired roles of pharmacy technicians were investigated as a possible barrier for pharmacists performing clinical services as medication therapy management (MTM), lack of sufficient staffing was reported to be of high influence. Few pharmacy technicians were trained to assist with MTM, but more than 70% of pharmacists would seek technical assistance with scheduling, billing and patient correspondence (USA).^21^

In order to prepare new cadres of mid-level pharmacy staff, a practice analysis was conducted to ensure that a new education program addressed workplace needs (South Africa).^22^ Another example of how research formed pharmacy technician role expansion, comes from the hospital setting. Department leadership and frontline staff identified the need for innovative pharmacy technician leadership roles. When developing those roles, leadership sought input from inside and outside the pharmacy department. Job descriptions and metrics were thus developed. Challenges in this project related to staff training and scalability of the model beyond the pilot setting (acute care). Besides firm ideas on the new pharmacy technician roles, this development has increased stakeholders (nursing, patients, physician) satisfaction with pharmacy services (USA).^23^

Research with regard to perceptions and attitudes about patient orientated pharmacy practice of pharmacy technicians was conducted.^24^ The objective was to obtain more insights into the professional behavior of pharmacy technicians within a USA practice setting. In the educational department changes have already occurred in Canada. Standards of practice for registered pharmacy technicians are developed by the provincial college of pharmacists (Ontario)^4^ and a national examination process is offered to pharmacy technicians by the Pharmacy Examining Board of Canada.^23^

The implementation of a successful practice model change is described to be availed by a sound operational strategy, excellent communication skills and the ability to navigate complex political issues, with a critical role for the pharmacist as organizational change leaders (USA).^3^, ^13^ A more applied description of what was done to make the most effective use of pharmacy staff resources to advance pharmacy services and optimize patient care in a USA setting, suggests that pharmacists provide patient centered care and certified, trained and competent pharmacy technicians perform ‘Tech-check-Tech’, medicine distribution functions that do not require clinical judgement, and other innovative functions.^11^ In order to reach that status in pharmacy care, the author recommends to learn (from precursors), share (best practices on current training programs), collaborate (developing advanced role model training programs), identify and ensure that this new level cadre is present in the departments.^11^ Another example is provided from Tanzania.^25^

Further, staffing and education are presented as important elements for effective change management involving pharmacy workforce roles. Harnessing the desire of staff to practice at a higher level is considered to be an important driver for role expansion (USA).^3^ Practicing at a higher level requires education. Nationally established standardized educational expectations and the introduction of rigorous entry-to-practice criteria are suggestions to address the raised questions on competency of pharmacy support workforce cadres (Canada).^1^ Depending on the anticipated extent of [possible] independent provision of services it is suggested that pharmacy support workforce cadres would need additional focused training to support the practical application of knowledge coupled with basic clinical skills. Continuous professional development (CPD) or workplace training may satisfy this need for focused training (Canada).^1^

Some available data beholds a warning. The Pharmacy Practice Model Initiative (PPMI) inspired the following statement, “*If pharmacists do not decide what their future practice model will be, others will decide for them. So be bold in planning for transformation of the pharmacy enterprise. Reach out internally and externally*” in the healthcare system to find allies (USA).^2^ From the rural environment, where shortages of pharmacists are an important issue, evidence is gathered on functions from the medical department (i.e. nurses) being up-skilled to fill the void in pharmacy-based functions (Australia).^5^ This task shifting by up skilling clinical staff from adjacent healthcare professions without any formalization, is also reported in Tanzania where it mainly occurs as a coping mechanism rather than a formal response to the workforce crisis.^25^

Concerns have also been voiced in the context of community pharmacy in the United Kingdom. The extension of the community pharmacist role, sought after by policy makers and pharmacy's representative bodies, involves medicine use reviews. These are often performed outside of the pharmacy. Absence of the pharmacist has an impact on pharmacy support staff and work processes. Consideration should be given to support staff and pharmacist's existing work obligations when developing extended roles. The provisions of adequate resourcing for the new (or old) services is needed to avoid well-intended improvisations by the staff (United Kingdom).^26^

The need to carefully consider readiness for change and good change management practice is further highlighted in this issue of the journal through case studies from Malawi, Singapore, South Africa, and the United Kingdom, which clearly document how these issues were addressed in country specific contexts.

## 5 Further examples of expanded roles of pharmacy technicians and other pharmacy support workforce cadres

The literature reports on a variety of extended pharmacy support workforce roles that have been studied in specific countries and practice contexts (mainly in Canada, the United Kingdom and USA). Many studies are exploratory in nature with a call for more research in the area.

Research on best possible medication history (BPMH) shows trained pharmacy technicians to obtain this history at hospitals as accurately and completely as pharmacists (Canada), while a recent meta-analysis of medication reconciliation approaches called for further studies to measure the impact of involving pharmacy technicians.^27^, ^28^ A study on pharmacy technician awareness of national medicine shortages aimed to enable pharmacy technicians to understand why shortages occur and how they can help ameliorate the effect of medicine shortages in their workplace. Pharmacy technicians proved a valuable resource for pharmacists in the management of medicine shortages and can work with pharmacists to perform operational and assessment tasks after a medicine shortage has been identified (USA).^12^

A review of multiple studies supported the practice of ‘Tech-check-Tech’ (TCT), a system in which pharmacy technicians check the correctness of prescription fulfilment by other pharmacy technicians, instead of the pharmacist. It demonstrated comparable accuracy and in some studies the increase in pharmacist time available for clinical activities was quantified. This overview even called for using the key elements of TCT to serve as a framework for development of future innovation in pharmacy technician roles (United Kingdom, USA).^11^

Economical outcomes are not used frequently in the conversation on pharmacy support workforce role expansion, even though it is essential in today's world of healthcare reform where pharmacy leaders seek to find ways to optimize patient care without increasing costs.^3^ A study that looked at using pharmacy technicians to aid in the completion of comprehensive medication review concluded that from an economic point of view return on investment for role expansion was valuable (USA).^29^

Supporting pharmacists to meet accreditation standards was researched in 2013. It showed five domains in which pharmacy technicians and other pharmacy support workforce cadres can play a role, practice management, patient counselling, patient care services, technology and quality improvement (USA).^30^ One of the most recent studies looked at the active role pharmacy technicians can have in the management of adherence programs, set up to improve medication adherence and reduce healthcare costs. The burden on community pharmacists running these programs was reduced (USA).^31^

## 6 The role of legislation

As the ideas on the extended role of pharmacy support workforce cadres became more clear, so has the call for legislation of the new objectives of the profession in the literature. This began in 2011 when a USA statement was made that *“all drug distribution activities should be performed by technicians in accordance with state law”*.^3^ In 2013, the PPMI again influenced the conversation. The authors conclude that the *“PPMI steers towards standard, uniform and accredited pharmacy technician education, combined with some form of licensure. To advance the role of the pharmacy technician, the PPMI recommends the requirement of certified technician”* (USA).^32^ In that same year, the ASHP guidelines not only talked about sufficient and adequately trained staff, but they also specify requirements for pharmacy technicians in the USA.^33^

Research is used to shape the process of legislation around supervision. As community pharmacist's roles extend into clinical and public health services, the roles and responsibilities of the pharmacy support team may need reconfiguration. Research on supervision is one of the possible sources to feed the department of health to possibly further change legislation around supervision without physical presence of a pharmacist.^34^ In the Canadian context pharmacy technicians are described in 2014 as increasingly becoming part of a regulated profession, allowing them to fulfil a greater role in both dispensing medications and patient care.^16^ In the USA the standardizing of education, training certifications and licensing requirements is seen as imperative in order for pharmacy support workforce cadres to be effective in the roles and responsibilities surrounding the provision of optimal patient care.^35^ Globally the situation is varied in this regard as noted through our survey data presented in this journal issue. Not all countries require registration of pharmacy support workforce cadres (examples of countries requiring the registration of some pharmacy support workforce cadres include Canada, Denmark, United Kingdom, USA, South Africa). Countries that do not require regulation for pharmacy support workforce cadres have legislation in place that sets the full responsibility of pharmacy practice under the supervision of the pharmacist.

## 7 Developing pharmacy support workforce leadership to further assist pharmacists in their role

In the 2012 literature, the importance of developing leadership roles within the pharmacy support workforce community emerged in the USA. In this context pharmacy technicians have been acknowledged as untapped resources, some have independently assumed informal leadership positions and some have been promoted to a formal leadership position (N.B. These positions have been particularly in the areas of stores, business and human resources management). The authors of this paper comment that it is important to take time in mentoring, educating, and training technician leaders to aid in developing their roles and further assisting the pharmacist in theirs.^23^

The PPMI from USA again influences the conversation. As a follow up of the PPMI, Stanford Hospital conducted a review of the existing pharmacy practice model. They identified opportunities for improvement and gaps to be filled by creating innovative leadership positions on the technician level, i.e. manager of clinical effectiveness and manager of professional development (USA).^13^

## 8 The importance of the pharmacy workforce to work together

From a review of the literature prepared for this commentary and through careful consideration of the case studies presented in this journal issue it is clear that the development of new roles and responsibilities for pharmacy support workforce cadres in specific country contexts have been most beneficial to the profession of pharmacy when pharmacists and pharmacy support workforce cadres have acted as a team to consider what is best in the local context. Since 2012, research on improving patient care does show this change in perception.

In a study on the sources of distracting stimuli and interruptions within the pharmacy department in the USA both pharmacists and pharmacy technicians were asked to give their insights.^36^ In addition, an exploration of views of pharmacist's and pharmacy technicians in the USA on a revalidation process for pharmacy professionals was conducted, leading to the result that a single model is not desirable.^37^ Other research has concentrated on stakeholder views of pharmacy activities which can/cannot be safely performed during the absence of a pharmacist. Participants were pharmacists, pharmacy technicians and other pharmacy support staff.^34^

In New Zealand, both pharmacists and pharmacy technicians were asked for their opinions on pharmacy technicians taking on an advanced checking role. Extra training was welcomed by both groups.^18^ Research on the perceptions of pharmacy technicians of their own work environment looked into elements such as recognition of organizational value, responsibilities, rate of pay, seeking equity among peers.^38^ To be able to get insights in the work engagement of pharmacy technicians and when the quality of the double-check process in the drug distribution system was assessed, the pharmacy team as a whole was involved to come up with an action plan to optimize the work processes.^39^

## 9 General conclusions

‘*It is not the strongest of the species that survives, nor the most intelligent, it is the one that is most adaptable to change*’

Charles Robert Darwin

In recent years, pharmaceutical services delivery has moved from a focus on medicines availability to an increasing need to deliver extended pharmaceutical clinical services to optimize patient health outcomes. As this change has unfolded, the role of pharmacists, technicians and other pharmacy support workforce cadres has evolved in different contexts and countries in an attempt to meet this need. The pharmaceutical literature has documented this evolution most notably in advanced economies (e.g. Canada, United Kingdom and USA), with many key insights of their experiences provided. These insights could be considered by other countries as they reflect on the ongoing evolution of pharmaceutical services delivery and the human resource requirements to meet this need.

Where pharmacists, pharmacy technicians and other pharmacy support workforce cadres work together in the context of their local environment, with a focus on improved patient care, optimal pharmaceutical services delivery can be provided.
